# Thermal Transport and Thermal Polarization of Water in the Supercooled Regime

**DOI:** 10.1021/acs.jpclett.4c02131

**Published:** 2024-09-18

**Authors:** Guansen Zhao, Fernando Bresme

**Affiliations:** Department of Chemistry, Molecular Sciences Research Hub Imperial College, W12 0BZ London, United Kingdom

## Abstract

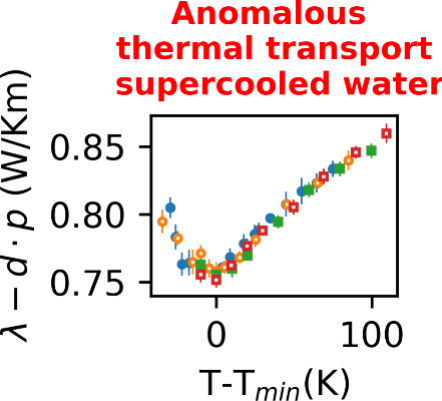

The behavior of water in the deep supercooled regime has attracted significant interest, motivated by the hypothesis of the second critical point of water. Previous studies indicated the existence of a water anomaly, characterized by a minimum in the thermal conductivity of water. Here, we employ nonequilibrium molecular dynamics computer simulation and the TIP4P/2005 water force field to investigate the thermal conductivity of supercooled water targeting four different isobars, 1, 200, 700, and 1200 bar. We demonstrate using NEMD simulations the existence of minima in thermal conductivity associated with the maximum isothermal compressibility and the minimum speed of sound in water, hence establishing a firm connection with the second critical point of liquid water. Moreover, we demonstrate that thermal gradients polarize supercooled water with a thermal polarization coefficient of several mV/K. We explain the thermal polarization effect using a theoretical formulation introduced recently that connects the thermal polarization effect to the isobaric thermal expansion coefficient.

**W**ater is the most common liquid on earth, and it holds immense significance in all living organisms, enabling biochemical and physicochemical processes. Moreover, water is a fascinating liquid, characterized by many anomalous properties that distinguish it from other liquids. The anomalies include the density maximum and minimum in the isothermal compressibility.^1,2^ The physicochemical behavior of supercooled water has attracted significant attention in the last decades. Glassy water forms under rapid cooling at rates exceeding 10^6^ K/s.^3^ However, nonglassy supercooled water can also be formed; e.g., it is commonly found in clouds and plays a vital role in the Earth-atmosphere system by absorbing and reflecting radiation.^4^

It has been shown that supercooled water displays an anomalous response with temperature, characterized by an increase of the isothermal compressibility and the isobaric heat capacity.^5,6^ To explain these anomalies, Poole et al. introduced the hypothesis of water polyamorphism. This hypothesis was tested using computer simulations of the ST2 water models and it was proposed the existence of a liquid–liquid phase transition (LLPT) between a high-density liquid and a low-density liquid, with the transition finishing at a liquid–liquid critical point.^7^ According to the LLPT hypothesis, the anomalous properties observed in supercooled water can be attributed to the fluctuations when the liquid approaches the critical point. Several studies, both experimental^8−11^ and simulation, including both empirical force field and density functional theory calculations,^12−15^ support the existence of the LLPT critical point in the deeply supercooled region of water. However, the LLPT hypothesis has attracted significant debate and discussions in the past few years.^14,16,17^

The intriguing question of whether water can possess a second critical point,^3^ combined with the practical applications related to water, has made supercooled water a subject of continuous interest since the 1970s. However, studying supercooled water in laboratory conditions is challenging due to its highly metastable nature and its tendency to form ice crystals, and computer simulations have emerged as a key tool for investigating supercooled water, especially at low temperatures. Recent experimental approaches have made progress in overcoming rapid ice nucleation enabling the study of the deeply supercooled region.^8,9^

Here, we investigate the thermal transport of supercooled water under explicit thermal fields. Specifically, we are interested in the variation of the thermal conductivity with temperature and the thermal polarization of supercooled water. Experimental measurements of water’s thermal conductivity have shown an unusual decreasing trend as the temperature approaches and falls just below freezing.^18^ However, the thermal conductivity behavior in the deeply supercooled region is still poorly understood. Recent theoretical analyses^19^ and computer simulations using the TIP5P^20^ and TIP4P/2005^21^ water reported the existence of minima in the thermal conductivity of supercooled water. The minimum appears at a temperature that varies with pressure^21^ and that aligns with the temperature at which the isothermal compressibility and the speed of sound feature a maximum and a minimum, respectively. The existence of the minimum can also be inferred from the Bridgman equation,^21−25^ λ_*B*_ = 2.8*k*_*B*_*v*^–2/3^*c*_*s*_, where λ_*B*_ denotes the thermal conductivity of water; *v* is the molecular volume; and *c*_*s*_ represents the speed of sound. Shortly after, another simulation study concluded there is no minimum thermal conductivity in supercooled TIP4P water models.^26^

The Green–Kubo approach is widely used to compute the thermal conductivity of fluids and materials. However, issues associated with the use of the Green–Kubo expression have been reported recently.^27,28^ Moreover, the existence of coupling effects between heat and polarization fluxes has been demonstrated,^29,30^ and following nonequilibrium thermodynamics, such a thermal coupling effect needs to be accounted for within a Green–Kubo formulation, as is done for example to compute the thermal conductivity of binary mixtures undergoing the Soret effect.^31^ Nonequilibrium simulations provide an ideal approach to quantify the thermal conductivity of molecular liquids since they account for additional thermal coupling effects, such as the thermal polarization effect.

In this work, we use state-of-the-art nonequilibrium simulations of the TIP4P/2005 model. Our simulations span long time scales (each run has a minimum duration of 200 ns), which are necessarry to ensure the appropriate sampling of the slow water dynamics and to reduce the statistical uncertainty of the computations. The nonequilibrium simulation method provides a direct route to quantity the water response to the explicit thermal field. We demonstrate that the thermal field polarizes supercooled water, and the polarization strength is proportional to the isobaric thermal expansion of water, following the predictions of a theoretical formulation introduced recently.^30^

We start our discussion by summarizing our simulation approach. We performed simulations with the TIP4P/2005 water force field,^32^ which is widely acknowledged as one of the most accurate rigid nonpolarizable models of water.^33^ Recent studies showed that this model reproduce numerous anomalous properties of water, such as the density maximum, the isothermal compressibility minimum, as well as dynamical properties, such as the viscosity.^34,35^ This model has also been widely employed to investigate the properties of supercooled water^36^ and ice polymorphs.^37^

We performed nonequilibrium molecular dynamics simulations using the boundary-driven method.^38^ In this method, thermal gradients are generated within the simulation box by thermostatting water molecules entering two different regions at the edges and middle of the simulation box, which are set to predefined hot, *T*_*H*_, and cold, *T*_*C*_, temperatures (see Figure 1). When the system reaches the stationary state, we compute the density and temperature profiles, which give access to the equation of state and thermal conductivity of water at different temperatures. Further details about the method are explained in the Supporting Information. The thermal conductivity (λ) was obtained using Fourier’s law
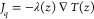
1by computing the local temperature gradient, ∇*T*(*z*) at position *z*. The heat flux along the direction of the thermal gradient is constant, and it was obtained using the continuity equation
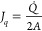
2where *Q̇* is the heat rate; *A* is the cross-sectional area of the simulation box in the direction perpendicular to the heat flux; and the factor of “2” accounts for the two heat fluxes generated in the simulation cell (see Figure 1). The heat rate can be obtained from the analysis of the energy exchanged at the hot and cold thermostats, which should be equal for a NEMD simulation with good energy conservation. We show in SI (Figure S1) that our method provides excellent energy conservation, and the heat rates obtained from the hot and cold regions agree within statistical uncertainty.

**Figure 1:**
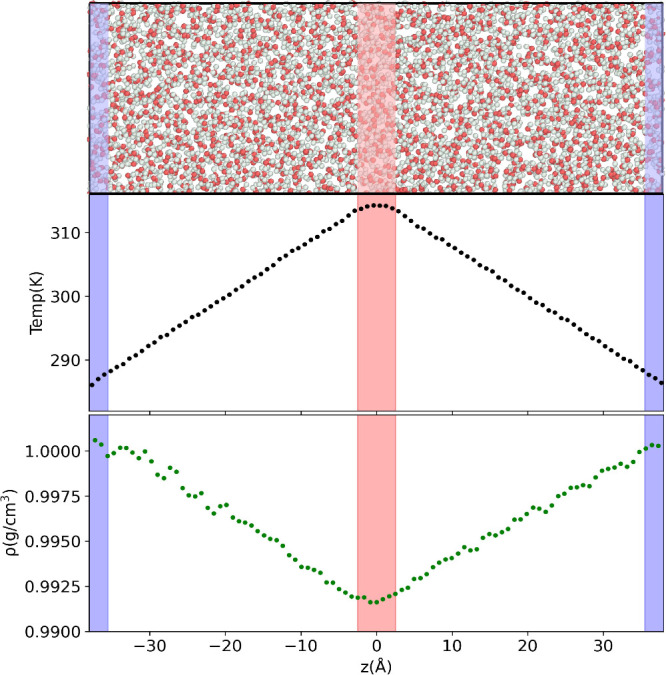
(Top): NEMD simulation setup for the simulation of water. White and red spheres denote H and O, respectively. (Middle) Example temperature and (Bottom) density profiles obtained from NEMD simulations. The thermostatting regions are represented by the red (hot) and blue (cold) areas.

We performed long simulations spanning at least 200 ns, and 100 ns were discarded to ensure the system reached the stationary state. We computed the density and temperature profiles along the direction of the heat flux. Additionally, we computed the charge density, ρ_*q*_(*z*), to obtain the electrostatic field, *E*_*z*_
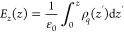
3induced by the thermal gradient and the thermal polarization coefficient^29,39,40^
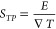
4We split the electrostatic field into its dipolar and quadrupolar contributions. These contributions define *S*_*TP*_ in a recent theoretical formulation of thermal polarization,^30^
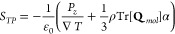
5where ρ is the molecular density; *P*_*z*_ is the dipolar contribution to the electrostatic field (see SI); Tr[**Q**_*mol*_] is the trace of the molecular quadrupole moment, **Q**_*mol*_; and α is the isobaric thermal expansion coefficient, which can be obtained from the equation of state:
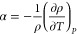
6A typical NEMD simulation involved 2100 water molecules, and the trajectories were integrated using LAMMPS (vs 3 Mar 2020).^41^

In addition to NEMD, we performed equilibrium simulations in the NPT ensemble to compute the speed of sound from the liquid’s isentropic compressibility and density. The SI provides full details of the simulation methods and additional equations employed to calculate dipolar and quadrupolar contributions, as well as data of the speed of sound, isothermal compressility, and isobaric thermal expansion.

We show in Figure 2 the equations of state (EOS) of the TIP4P/2005 water for three isobars investigated in this work, 1, 700, and 1200 bar. The EOS was obtained using both equilibrium and nonequilibrium simulations. To represent the NEMD data we used the pairs of density and temperature generated in the NEMD simulation box. The NEMD simulations involved different windows with average temperatures targeting different temperature intervals (see SI for details and numerical results). The blue lines in Figure 2 represent the NEMD data using a running average over 50 points. At low temperature the fluctuations of EOS depend strongly on the bin size used (see Figure S4 in the SI), and the running average allows us to obtain the EOS in this low temperature regime. We note that the NEMD and equilibrium simulations require significant sampling at low temperatures to obtain convergence. As indicated in ref (21), getting convergence in the equilibrium simulations requires times approaching 100 ns.

**Figure 2:**
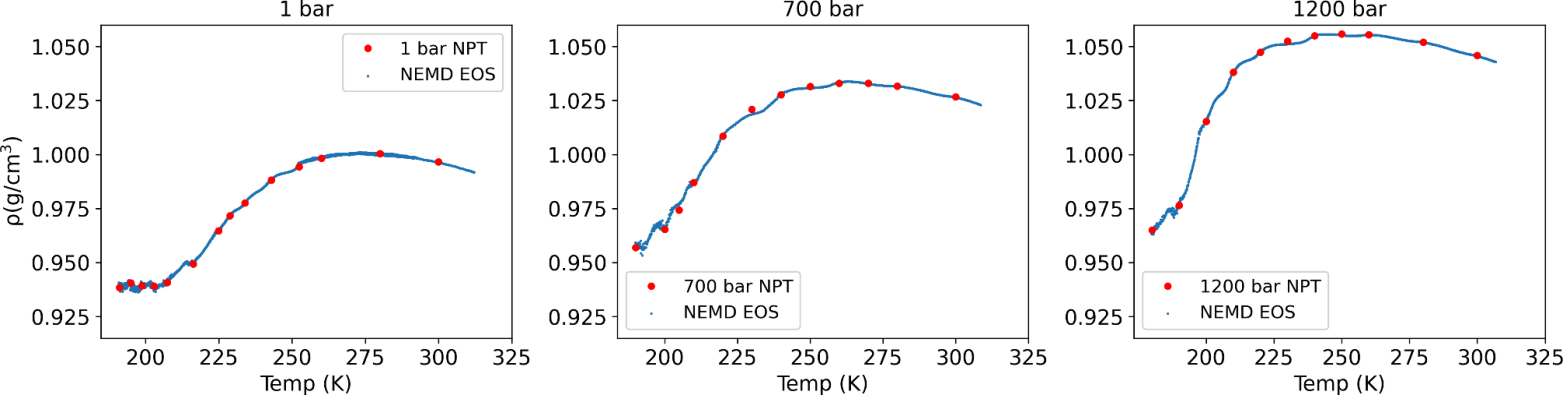
Equations of state from equilibrium NPT simulations (red dots) at three isobars and various NEMD windows (blue) with moving averages applied to reduce noise (see SI). Panels from left to right represent data for 1, 700, and 1200 bar.

In the deeply supercooled region (≤210 K), the supercooled liquid features very slow dynamics as the system approaches the glass transition temperature.^3,42,43^ Hence, to ensure our systems were well-equilibrated, we analyzed the pressure of the constant volume NEMD simulations in 10 ns time intervals (see Figure S2 in the SI). Our data show good convergence at high temperatures. However, there is clear dynamic slow down in the deeply supercooled region, *T* < 210 K at 1 bar (see Figure S2 in the SI). In that region, the system requires ∼50–100 ns to reach the pressure consistent with the average temperature and density profile of the system.

The high level of consistency between the two sets of equations of state data, NPT and NEMD (see Figure 2), supports the local equilibrium hypothesis^31^ at the thermodynamic conditions under investigation. Moreover, the good agreement between equilibrium and NEMD results confirms the simulations are sufficiently long to achieve convergence. Also, our results show good agreement with data reported in previous works.^21,42^ One noticeable feature of the EOS represented in Figure 2 is the appearance of an inflection point in the density at low temperatures.

Figure 3 shows the thermal conductivity for the three isobars represented in Figure 2 and also the 200 bar isobar. We analyzed the convergence of the thermal conductivity with time. As shown in the panels of Figure S3 in the SI, the thermal conductivity converges quickly at temperatures higher than 220 K. However, at lower temperatures the convergence is slow, and we require at least 100 ns to eliminate the drift in the thermal conductivity (see 195 K system at 1 bar pressure in Figure S3). This observation supports the conclusions of ref (21) where it was noted that long equilibrium simulations were required to ensure the convergence of the results. As shown in Figure 3 (left), the thermal conductivity displays a minimum in the supercooled region for all the pressures we have investigated. The temperature where thermal conductivity reaches its minimum (*T*_*min*_) decreases with rising pressure. In addition, the *T*_*min*_ for 1 bar at 220 K is close to the temperature corresponding to the minimum in the isobaric thermal expansion coefficient, the minimum in the speed of sound and the maximum in the isothermal compressibility (see the markers in Figure 3, left). This correlation is also observed for the higher presure isobars (700 and 1200 bar), as shown in Figure S5 in the SI. However, *T*_*min*_ for these higher pressures is notably low, posing a considerable challenge in simulating the low-temperature states. This prompted our analyses of an intermediate isobar, 200 bar, to confirm the existence of the minimum in the thermal conductivity. Our results feature a clear minimum at *T*_*min*_ ∼ 215 K and hence shifted to lower temperatures with respect to the standard pressure result.

**Figure 3:**
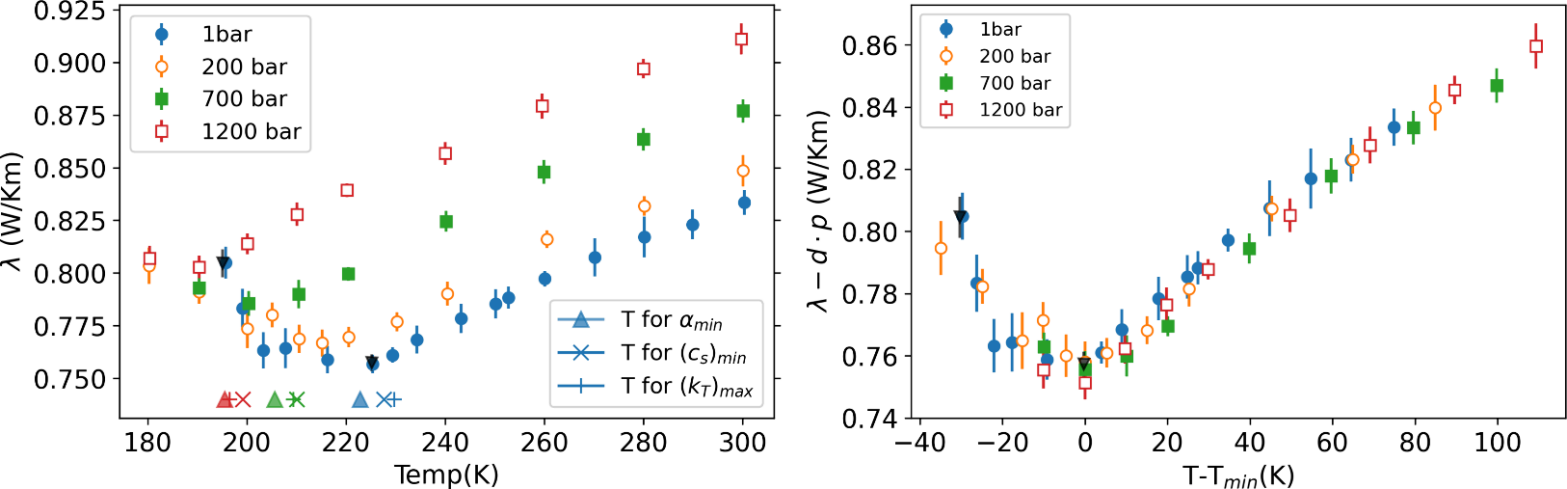
(Left): Average thermal conductivity with error bars calculated from 100 to 200 ns. The temperatures for the minimum thermal expansion coefficient, minimum speed of sound in water, and maximum isothermal compressibility at 1 bar pressure are shown with △, ×, and + symbols, respectively. (Right): The linear scaling corrected thermal conductivity with a factor *d* = 0.0429 mW K^–1^ m^–1^ bar^–1^, as a function of *T* – *T*_*min*_. The inverted black trinagle in the left and right panels represents NEMD thermal conductivities at 1 bar, obtained with smaller gradients (∼0.36 K/m), about half of the thermal gradient employed in the other computations (vs ∼0.74 K/m).

We plot in Figure 3 (right) a correlation plot that represents our results taking into account the linear dependence of the approximate thermal conductivity with pressure. We follow ref (21) and rescale the data by the temperature and thermal conductivity at the minimum. Figure 3 (right) shows that the data align very well into a single “master” curve. The linear dependence in the pressure quantified through the parameter *d* (see Figure 3, right) is similar to the value obtained before using Green–Kubo computations. The rescaling into a single master curve highlights the thermodynamic connection of the minimum of thermal conductivity and the thermodynamic anomalies. This is further shown in Figure 3 (left) where we have indicated the location of the extrema of the isobaric thermal expansion, isothermal compressibility, and the speed of sound, showing the extrema in these properties aligns very well with the location of the thermal conductivity minimum. We have collated in the Supporting Information numerical data for these three properties as a function of temperature and pressure.

The agreement between the NEMD and equilibrium equations of state (see Figure 2) supports the local equilibrium and linear response for the thermal gradients employed here. To verify this point further we performed additional simulations of two state points at 1 bar pressure, one point at low temperature and the other in the region corresponding to the minimum in the thermal conductivity, by using a smaller thermal gradient. The data in Figure 3 and Table S1 agree with results obtained with the higher thermal gradient, confirming our computations are in the linear regime.

Recently, Espinosa et al. reported simulations of the TIP4P/ICE water model. They conclude that the 400 bar isobar for this model accurately reproduces the experimental isothermal compressibility of water and the liquid equation of state. Furthermore, a LLPT was inferred from the simulation studies.^44^ Hence we performed additional NEMD simulations of the TIP4P/Ice model at 400 bar to search for a minima in the thermal conductivity. Our thermal conductivities are shown in Figure S7 in the SI along with the data for the TIP4P/2005 model at 1 bar. The thermal conductivity of TIP4P/Ice is noticeably larger, a result connected to the higher pressure in these simulations. The conductivities are similar to the values obtained with the TIP4P/2005 model at 700–1200 bar. Our simulations support the existence of a thermal conductivity minimum in TIP4P/Ice in the interval 210–220 K, hence in the region corresponding to the maximum in the isothermal compressibility reported in ref (44). These results support the thermodynamic origin of the thermal conductivity minimum and the anomalous heat transport of supercooled water, which is associated with the presence of an LLPT.

Previous computer simulations and nonequilibrium thermodynamics studies showed that a heat flux induces the polarization of polar fluids such as water,^29,45^ leading to substantial electrostatic fields for large thermal gradients, achievable in nanoscale heating experiments.^46^ The strength of the thermal polarization can be quantified using the thermal polarization coefficient, *S*_*TP*_ (eq 4). Recently a microscopic equation of the *S*_*TP*_ of water was proposed. This equation incorporates dipolar and quadrupolar contributions (see eq 5). the quadrupolar contribution to the thermal polarization coefficient is proportional to the isobaric thermal expansion coefficient, α. As shown in Figure 2, α features important changes in the supercooled regime, including a change in sign and a minimum. Based on eq 5 and α < 0 in the deeply supercooled regime, we hypothesize that *S*_*TP*_ could feature an enhancement in the supercooled region, as well as a nonmonotonic dependence of the quadrupolar contributions to the thermal polarization of water. We test this hypothesis in the following.

We computed the thermal polarization coefficient from the thermally induced electrostatic fields, using eqs 3 and 4. Further, we have computed the dipolar and quadrupolar contributions using eqs 7–9 in the Supporting Information. The *S*_*TP*_ coefficient can be obtained from −*dϕ*/*dT*, where ϕ is the electrostatic potential in the simulation box, calculated using eq 10 in the SI. Figure 4 shows the thermal polarization coefficient *S*_*TP*_ obtained from the NEMD simulations at 1, 700, and 1200 bar. In addition to the total *S*_*TP*_ we show the dipolar and quadrupolar contributions.

**Figure 4:**
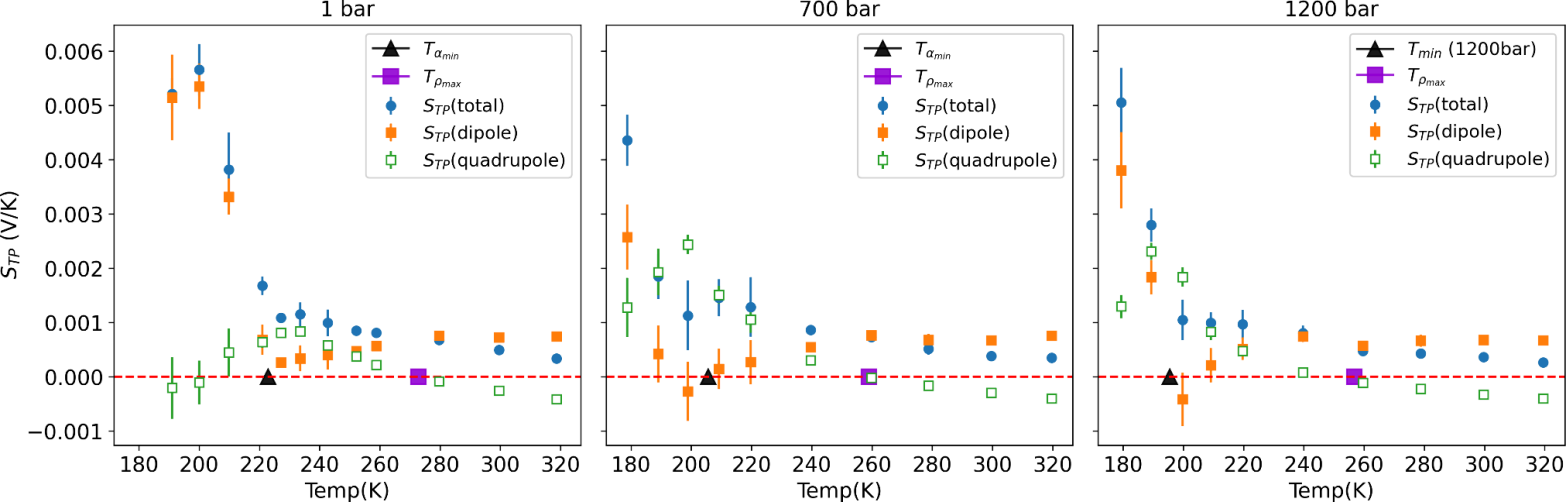
Thermal polarization coefficient and dipolar and quadrupolar contributions. The markers represent the temperature of density maxima (squares) and thermal expansion minima (triangles) for the 1, 700, and 1200 bar systems.

The dipolar contribution is nearly constant at high temperatures, while the quadrupolar term varies with temperature following the temperature dependence of the isobaric thermal expansion coefficient. It is negative above the α = 0 and positive below. The cancellation of the quadrupolar contribution, *S*_*TP*_(*quadrupole*) = 0, agrees with the location of the density maximum at the different pressures. At temperatures below the density maximum where α < 0 the quadrupolar contribution becomes positive and does also feature a maximum at the temperature corresponding to the minimum in the isobaric thermal expansion (see Figure 4). This complex polarization behavior is reflected in the total thermal polarization of supercooled water. We infer from our simulations that the thermally induced polarization varies nonmonotonically with temperature featuring local maxima or inflection points that reflect the thermal expansion minima appearing in the region corresponding to the LLPT thermodynamic anomalies.

The thermal polarization effect of supercooled water is rather strong. We find coefficients *S*_*TP*_ ∼ 1 mV/K and larger ∼5 mV/K, at standard pressure. These large polarization effects emerge from the enhancement of the quadrupolar contributions, and particularly the dipolar term at low temperatures (see Figure 4, left). The magnitude of the thermal polarization coefficient is similar to the values reported for TIP4P/2005 water at conditions near the primary critical point^47^ and indicates that the application of thermal gradients should induce a significant polarization in supercooled water.

In summary, we have demonstrated, using nonequilibrium molecular dynamics simulations, the existence of minima in the thermal conductivity of supercooled water modeled with the TIP4P/2005 and TIP4P/Ice models. For TIP4P/Ice the thermodynamic conditions chosen very accurately reproduce the experimental isothermal compressibility of water.^44^ The minima in the thermal conductivity correlate with the temperature of maximum compressibility, minimum isobaric thermal expansion, and minimum in the speed of sound. Our data therefore support the thermodynamic origin of the thermal conductivity minimum and the connection of this water anomaly to the LLPT of supercooled water. Furthermore, we have shown that the application of a thermal gradient polarizes supercooled water, inducing an electrostatic field in the liquid. Following nonequilibrium thermodyamics, this nonequilibrium coupling effect influences the thermal transport of water, making it necessary to include this effect in computations of thermal transport properties, such as the thermal conductivity. The use of explicit thermal gradients via nonequilibrium computer simulations provides a direct route to include all these nonequilibrium effects.

We showed that the extrema in the isobaric thermal expansion induces a nonmonotonic dependence of the thermal polarization response. The polarization features an enhancement around temperatures corresponding to the thermal conductivity minimum. The microsocopic origin of the enhancement is connected to the electrostatic quadrupolar contributions and the dipolar terms in the deeply supercooled regime. In this regime, the thermal polarization response is similar in magnitude to that observed in previous simulation studies of supercritical water. Based on our calculations, we estimate that thermal fields achievable at the nanoscale, 10^6–8^ K/m, could induce thermal polarization fields of ∼10^3–6^ V/m in supercooled water. The present study indicates that these thermal coupling phenomena are important in defining the thermal transport properties of water. Further investigations should help to advance our understanding of the heat transport mechanism of water under supercooled conditions.

## Supporting Information Available

Computer simulation details, time dependence of thermophysical properties, details on the thermal polarization calculations and additional data on the temperature dependence of the isobaric thermal expansion coefficient, isothermal compressibility, and the speed of sound and thermal conductivity minimum of TIP4P/Ice (PDF)
