# Supplementary material for: Thermal Transport and Thermal Polarization of Water in the Supercooled Regime
Source: J Phys Chem Lett. 2024 Sep 18;15(38):9774–9. doi: 10.1021/acs.jpclett.4c02131 (PMC11440598; doi:10.1021/acs.jpclett.4c02131)
Supplement: Supplementary file 1 — jz4c02131_si_001.pdf [file jz4c02131_si_001.pdf]

Supporting Information: Thermal Transport and Thermal Polarization of Water in the Super-Cooled Regime

Guansen Zhao and Fernando Bresme*

Department of Chemistry, Molecular Sciences Research Hub Imperial College, W12 0BZ, London, United Kingdom

E-mail: f.bresme@imperial.ac.uk

Phone: +44 207 594 5886

1. Simulation Setup

The NEMD simulation box dimensions were $\{L_x : L_y : L_z\} = \{1 : 1 : 3\}$, where $L_x \approx 25$ Å. There are two thermostatting regions in the simulation box: two cold regions at the edges of the z-axis and a hot region at the center. Each region has a thickness of 0.5 nm. Consequently, two heat fluxes with equal magnitudes build up in opposite directions along the z-axis between the hot and cold regions. The magnitude of the heat flux depends on the temperature difference between the hot and cold regions (T_{hot} and T_{cold}). The Lennard-Jones interaction were calculated in full using the particle-particle particle-mesh (PPPM) method.[S1] Additionally, the PPPM variant[S2] of the Ewald summation was applied to account for long-range Coulombic interactions.

To setup the NEMD simulations, we started with equilibrium computations using the isothermal-isobaric (NPT) ensemble at the intended pressure and a temperature equal to

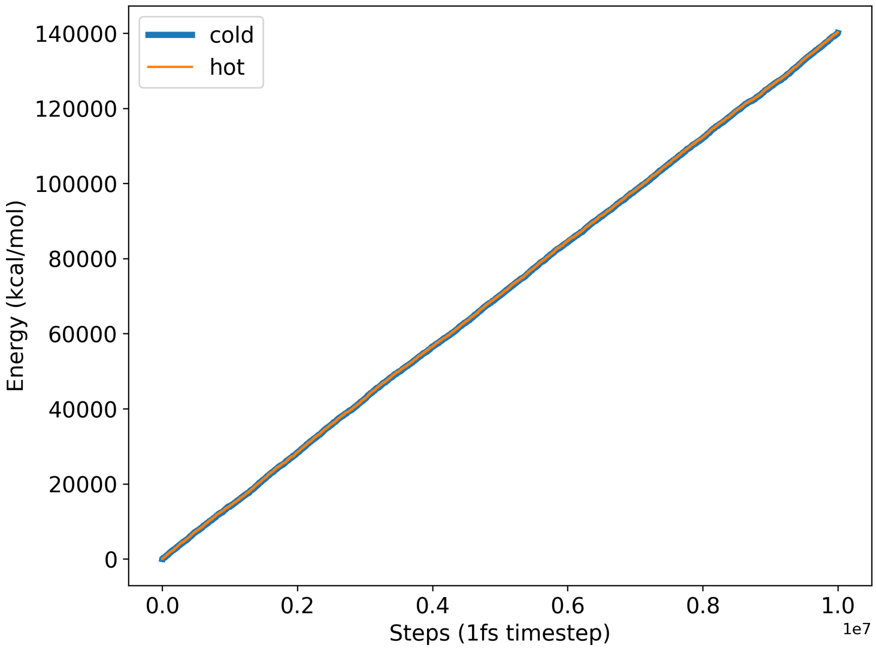

Figure S1: Energy conservation check for the NEMD simulation, where the blue and orange lines represent the energy exchange at the cold and the hot thermostatting regions versus steps, respectively.

the average of T_{hot} and T_{cold} for at least 1 μs with a 1 fs timestep. Using the NPT simulations results, we calculated the fluctuation properties (α: thermal expansion coefficient and k_T: isothermal compressibility) with Gromacs (vs. 2021.3-gpu).[S3] These fluctuation properties were calculated by averaging over every 200 million steps (200 ns simulation time). The thermal expansion coefficient and isothermal compressibility, $k_T = -(1/V)(\partial V/\partial P)_T$, were computed using the fluctuation relations in Eqn. (1) and (2), respectively.[S4]

$$\langle \delta V \delta H \rangle_{NPT} = k_B T^2 V \alpha, \tag{1}$$

$$\langle \delta V^2 \rangle_{NPT} = V k_B T k_T, \tag{2}$$

where V is the volume, H is the enthalpy, T is the temperature, k_B is the Boltzmann constant, and $\delta \mathcal{P} = \mathcal{P} - \mathcal{P}_{ens}$, where "ens" indicates the ensemble average of propery $\mathcal{P}$. The speed of

sound was calculated using:

$$c_s = \sqrt{\frac{C_P}{C_V}\frac{1}{\rho k_T}}, \tag{3}$$

where C_P and C_V represent isobaric and isochoric heat capacity; ρ denotes the mass density. The average density from the NPT simulations system was used to change the volume of the simulation box to achieve the target pressure. Next, we conducted NEMD simulations for a minimum duration of 200 ns with 1 fs timestep and thermostatting regions. Figure S1 shows a typical plot illustrating the time depend energy exchanged at the hot and cold thermostats. The agreement between the energy in these thermostats demonstrates excellent energy conservation.

Each production run involved at least three replicas initialized with different random number seeds. The simulation box was divided into 100 layers for the calculation of the local temperature, density, and pressure profiles. The obtained results at all the targeted temperatures and pressures are listed in Table S1. Following the NEMD simulations, the trajectories of the last 10 ns (190-200 ns), which were computed every 100 steps, were utilized to obtain the electrostatic properties for the thermopolarization calculations.

We show in Figure S2 and S3 the time evolution of pressure and thermal conductivity, respectively, at 1, 700, and 1200 bar pressures. The simulations feature slow dynamics at low temperatures, requiring a longer time for the system to equilibrate. Based on these results, we decided to exclude the data of the first 100 ns from the production run.

Begin of Table										
P_{target}	T_{target}	Time	Temp	T_{hot}	T_{cold}	ρ	P	P_{err}	λ	λ_{err}
0.99	195	480	195.7	209.4	180.6	0.941	21.6	66.1	0.805	0.0076
0.99*	195	280	195.1	202.2	187.9	0.941	13.3	27.2	0.802	0.0067
0.99	200	200	199.1	213	184.5	0.939	11.6	51.3	0.783	0.0092
0.99	203	200	203.4	217.4	188.5	0.939	8.7	67.8	0.763	0.0086
0.99	207	200	207.8	221.9	193	0.941	-10.3	71.4	0.764	0.0094
0.99	215	200	216.3	230.8	201.7	0.949	-6.8	40.5	0.759	0.0065

Continuation of Table										
P_{target}	T_{target}	Time	Temp	T_{hot}	T_{cold}	ρ	P	P_{err}	λ	λ_{err}
0.99	225	200	225.4	239.5	210.4	0.965	-8.8	15.8	0.757	0.0043
0.99*	225	180	225.2	232.3	217.7	0.964	-27.1	14.9	0.755	0.0039
0.99	230	200	229.4	243.3	214.5	0.972	-1.1	16.8	0.761	0.0036
0.99	235	200	234.3	248.5	219.3	0.978	-12.7	10.7	0.768	0.0065
0.99	243	200	243.2	257.5	228.3	0.988	15.8	5.2	0.778	0.007
0.99	252	200	252.8	267	238.1	0.994	8.6	12.8	0.788	0.0053
0.99	250	200	250.2	264.5	235.5	0.991	-7.9	13.9	0.785	0.007
0.99	260	200	260.1	274.4	245.7	0.996	-10	11.6	0.797	0.0036
0.99	270	200	270.1	284.3	255.6	1.001	25	9.4	0.808	0.009
0.99	280	200	280.2	294.3	265.7	0.999	-3.8	7.7	0.817	0.0097
0.99	290	200	289.9	304.2	275.8	0.999	22.1	7	0.823	0.0071
0.99	300	200	300.3	314.3	285.8	0.997	12.8	6	0.834	0.006
0.99	320	200	320.2	334.1	305.8	0.989	6.9	5.9	0.841	0.0067
0.99	340	200	340	354.2	325.9	0.978	0.1	6.6	0.84	0.0091
0.99	360	200	360.4	373.8	345.9	0.965	3	6.5	0.838	0.0088
0.99	380	200	379.9	394.1	365.8	0.95	-1.9	7.1	0.824	0.0067
0.99	400	200	400.1	414.2	385.8	0.933	-4.6	6.9	0.811	0.009
200	180	200	180.3	194.4	165.7	0.943	254.8	15.4	0.803	0.0086
200	190	200	190.4	204.3	175.6	0.941	226.6	30.3	0.791	0.0057
200	200	200	200.1	214.3	185.7	0.945	156.6	51.2	0.774	0.0091
200	205	200	205.1	219.5	190.6	0.948	218.9	37	0.78	0.0059
200	210	200	210.6	224.6	195.7	0.954	164.5	49.9	0.769	0.0068
200	215	200	215.2	229.5	200.7	0.962	200.1	41.3	0.767	0.0064
200	220	200	220.5	234.6	205.6	0.971	182.1	34.6	0.77	0.0048
200	230	200	230.3	244.6	215.7	0.987	213.9	29.2	0.777	0.0045
200	240	200	240.5	254.5	225.6	0.998	212	24.2	0.79	0.0056
200	260	200	260.5	274.4	245.6	1.009	209.6	14.5	0.816	0.0041

Continuation of Table										
P_{target}	T_{target}	Time	Temp	T_{hot}	T_{cold}	ρ	P	P_{err}	λ	λ_{err}
200	280	200	280.2	294.3	265.7	1.01	207	10.2	0.832	0.0046
200	300	200	300.1	314.2	285.8	1.006	206.1	8.5	0.849	0.0074
200	320	200	320	334.2	305.8	0.998	206.2	8.1	0.855	0.0086
200	340	200	340	354	326	0.988	207.8	7.9	0.854	0.0055
200	360	200	360.1	373.9	345.8	0.975	199	9.4	0.846	0.0058
200	380	200	380	394.1	365.8	0.961	200.1	9.6	0.837	0.0063
200	400	200	400.1	414.2	385.8	0.944	200.9	9.8	0.831	0.004
690.85	190	250	190.4	204.6	175.8	0.957	672.8	31	0.793	0.0045
690.85	200	250	200.3	214.5	185.8	0.965	663.7	37.2	0.786	0.0059
690.85	210	250	210.5	224.6	195.8	0.987	660	25.4	0.79	0.0067
690.85	220	200	220.5	234.6	205.6	1.009	701.6	14.5	0.8	0.0032
690.85	240	200	240.2	254.4	225.6	1.028	705.6	3.8	0.825	0.0049
690.85	260	200	260	274.2	245.7	1.034	709	2.8	0.848	0.0057
690.85	280	200	280	294.2	265.9	1.032	697.8	1.8	0.863	0.0054
690.85	300	200	300.1	314	285.9	1.027	700.7	1.3	0.877	0.0055
690.85	320	200	319.8	334.1	305.9	1.019	698.6	2.8	0.888	0.0063
690.85	340	200	340	353.9	325.9	1.008	696.4	1.8	0.885	0.0056
690.85	360	200	359.8	374	345.9	0.996	694.4	1.2	0.88	0.0088
690.85	380	200	379.8	393.9	366.1	0.983	689.1	0.9	0.873	0.0089
690.85	400	200	399.9	414	385.8	0.968	693.3	1.4	0.86	0.0088
1184.31	180	250	180.4	194.3	165.8	0.965	1180.1	41.5	0.807	0.006
1184.31	190	200	190.4	204.5	175.8	0.976	1220.5	52.5	0.803	0.0054
1184.31	200	200	200.2	214.5	185.9	1.015	1180.2	52.4	0.814	0.0049
1184.31	210	200	210.2	224.5	195.6	1.038	1199.5	33.6	0.828	0.0056
1184.31	220	200	220.2	234.3	205.7	1.047	1183.5	10.4	0.839	0.0033
1184.31	240	200	240	254.4	225.8	1.055	1194.7	4.5	0.857	0.0054
1184.31	260	200	259.5	274.2	245.9	1.056	1192.2	2.9	0.879	0.0059

Continuation of Table										
P_{target}	T_{target}	Time	Temp	T_{hot}	T_{cold}	ρ	P	P_{err}	λ	λ_{err}
1184.31	280	200	280	294	265.8	1.052	1190.8	2.1	0.897	0.0046
1184.31	300	200	299.7	313.9	285.9	1.046	1191	2.3	0.911	0.0073
1184.31	320	200	319.7	333.9	305.9	1.037	1189.3	1	0.914	0.007
1184.31	340	200	339.9	353.8	326	1.027	1189.6	1.9	0.92	0.0066
1184.31	360	200	359.9	374.1	346.1	1.016	1188	2.6	0.917	0.0077
1184.31	380	200	379.4	393.9	366	1.003	1186.5	1.9	0.907	0.0064

Table S1: Results of the NEMD simulations. The pressures are in bar, temperature in K, thermal conductivity (λ) in $W/(Km)$ and densities are in the units of g/cm^3. The "Time" column represents the total simulation time in nanoseconds. P_{err} and λ_{err} are the standard deviations calculated from the replicas in the production runs. The simulation time data with asterisk symbols at 195 and 225 K, 1 bar represent the simulation times for NEMD runs with smaller gradients 0.36 vs 0.74 K/m for the rest of the symbols. These simulations for the small thermal gradients were started using as starting point, the final configurations obtained from computations performed with larger thermal gradients.

End of Table

2. NEMD equation of state

For the calculation of the NEMD equation of state plot (Figure 2) in the main text, we applied moving averages with large window sizes (50) to reduce the noise associated to the data binning. To compute the NEMD EOS data, we took the local temperature and density of each bin as a data point for all the NEMD replicas at all temperatures. Then we combined all the data points, rearranged them, and plotted. The results involve more than 5000 points in the EOS data set, which requires a larger moving average window size to reduce noise. In Figure S4, we show the raw EOS data at 1 bar pressure, and its running averaged results with the window size of 5, 20 50. As the window size increases, our NEMD EOS data match very well the equilibrium NPT simulated results.

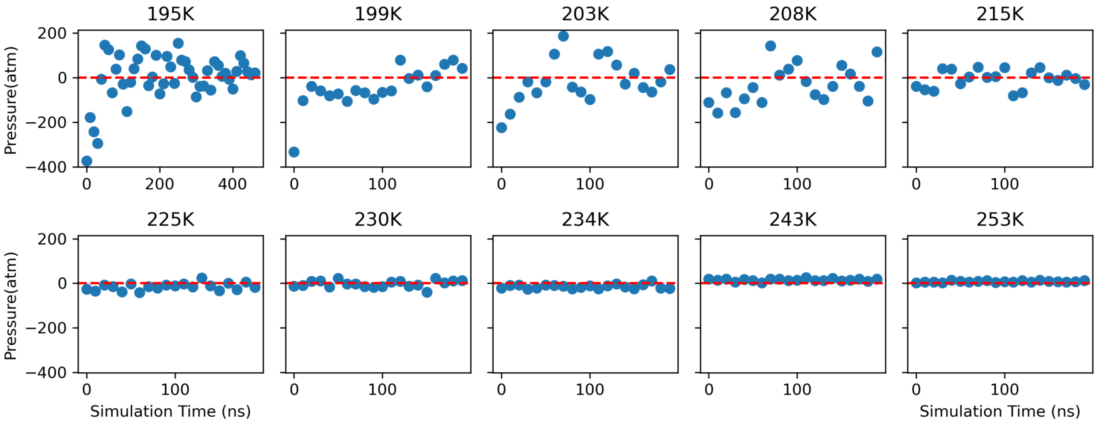

(a) 1 bar

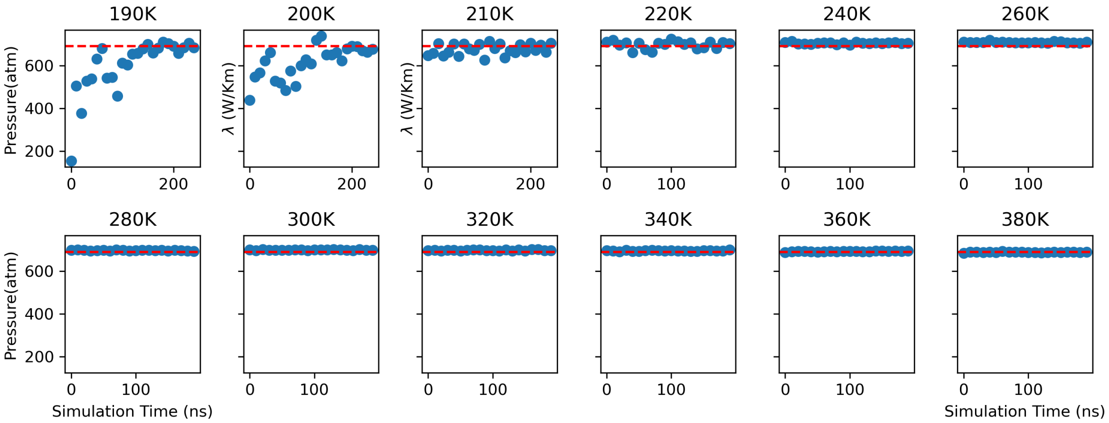

(b) 700 bar

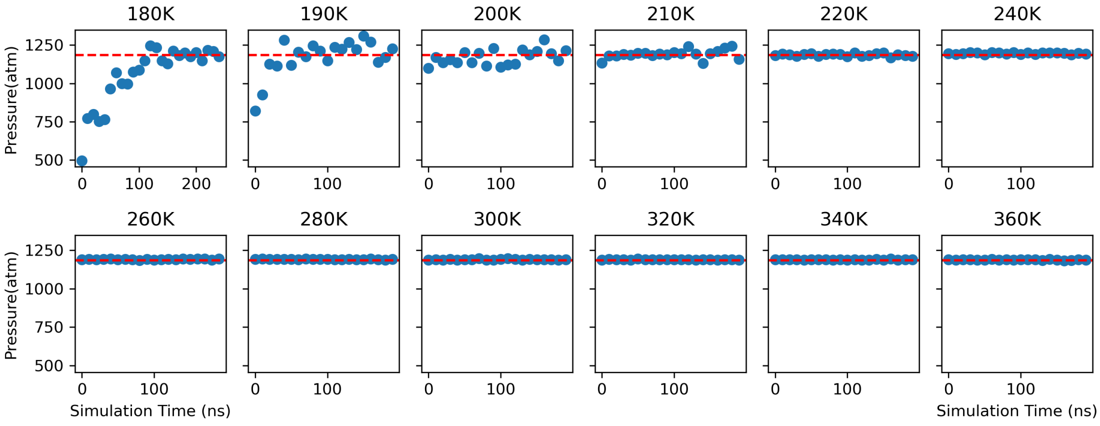

(c) 1200 bar

Figure S2: Time evolution of the pressure from 0-200 ns for the NEMD at different temperatures at the three isobars. (a)1bar 195-253K, (b)700bar 190-380K, (c)1200bar 180-360K.

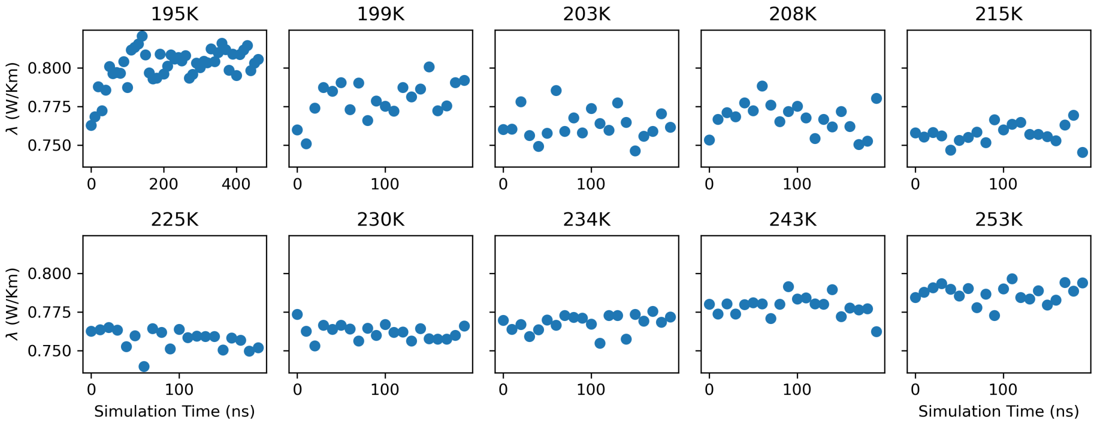

(a) 1 bar

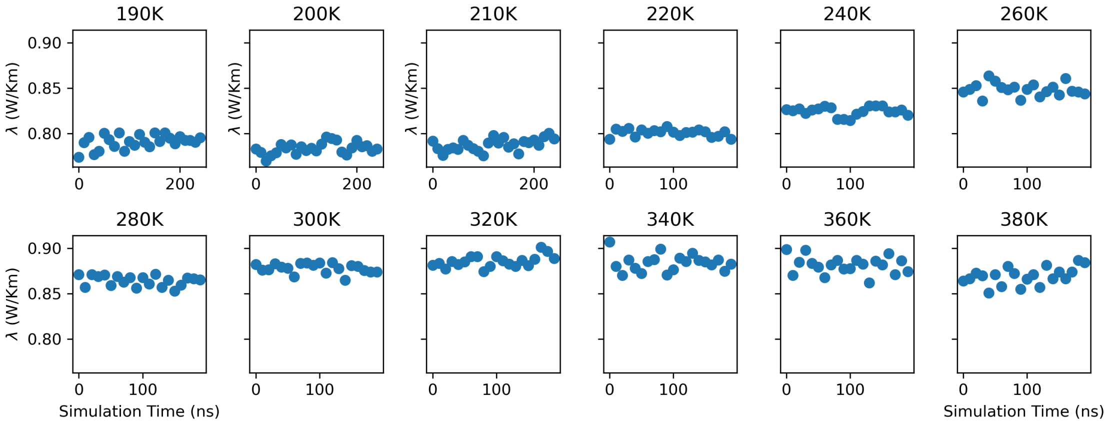

(b) 700 bar

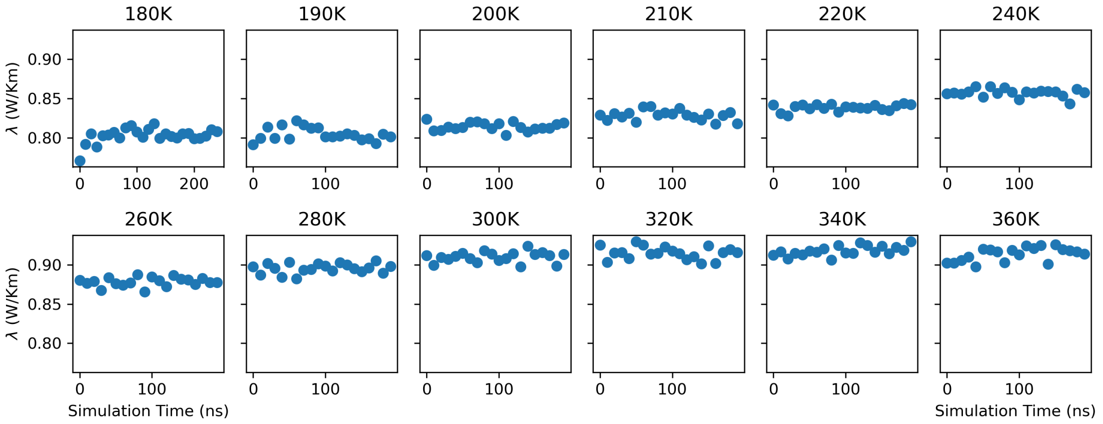

(c) 1200 bar

Figure S3: Time evolution of the thermal conductivity from 0-200 ns for the NEMD simulations at the three isobars.

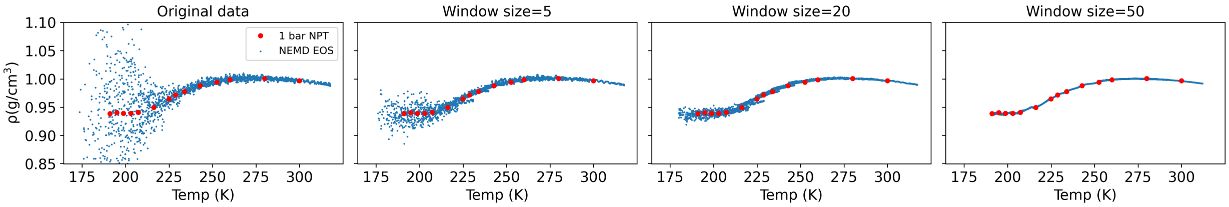

Figure S4: Equation of state from equilibrium NPT simulations (red dots) and NEMD runs (blue) with different moving average window sizes (from left to right: 0, 5, 20, 50) applied. All the data correspond to the 1 bar isobar, and the NEMD EOS are obtained from 100-200 ns simulations.

3. Thermal conductivity and polarization calculations

In order to calculate the thermal conductivity λ of water in the supercooled region, we used Fourier's Law,

$$\lambda = -\frac{J_q}{\nabla T}, \tag{4}$$

where ∇T denotes the thermal gradient, and J_q the heat flux. From the change in the kinetic energy associated to the thermostatting process. We emploted the CSVR thermostat.[S5] The momentum of the whole box was reset every timestep.

$$J_q = \pm\frac{\langle \Delta K \rangle}{\langle 2A\delta t \rangle}. \tag{5}$$

In Eqn. (5), ΔK represents the energy enters (+) or leaves (-) the thermostatting regions. A denotes the cross-sectional area of the simulation box, which is perpendicular to the direction of the thermal gradient, and δt is the timestep of the simulation. To quantify the thermal gradient precisely, we performed NEMD runs with a narrow temperature range of 40 K. A linear regression was employed to fit the local temperature profile close to the average temperature. Subsequently, the average temperature gradient was determined from the slope of the fitted line and the conductivity can be computed for each window.

For the polarization calculations, we calculated the electrostatic field E, using the charge density $\rho(z)$[S6,S7]:

$$\rho(z) = \frac{1}{L_x L_y}\left\langle \sum_{i}^{N_{atoms}} q_i \delta(z - z_i) \right\rangle. \tag{6}$$

In Eqn. (6), L_x and L_y are the lengths of the simulation box in the x and y directions, perpendicular to the direction of the heat flux, z. The term enclosed in the angular brackets represents the ensemble average of the summation running over all charged atoms, with q_i and z_i representing the charge and z-coordinate of the ith atom. For the TIP4P/2005 water model, the charged sites consist of the hydrogen ($q_H = 0.5564e$) and dummy atoms ($q_M = -1.1128e$).[S8] The dipolar, P_z, and quadrupolar, Q_{zz} contributions were calculated using[S7,S9,S10]:

$$\rho(z) = -\frac{\partial}{\partial z} P_z(z) + \frac{\partial^2}{\partial z^2} Q_z z(z) + \cdots\,, \tag{7}$$

$$P_z(z) = \frac{1}{L_x L_y}\left\langle \sum_{m}^{N_m} \delta(z - z_m) \sum_{i \in m}^{N_{charges}} q_{i,m} z_{i,m} \right\rangle, \tag{8}$$

$$Q_{zz}(z) = \frac{1}{L_x L_y}\left\langle \sum_{m}^{N_m} \delta(z - z_m) \frac{1}{2} \sum_{i \in m}^{N_{charges}} q_{i,m} {z_{i,m}}^2 \right\rangle. \tag{9}$$

We also calculated the electric potential by integrating the z component of the electric field:

$$\phi(z) = -\int_0^z E_z\, dz, \tag{10}$$

and in this way, S_{TP} can be computed with the first derivative of the electric potential as a function of temperature, as discussed in the main text.

4. Additional results for the correlation of the minimum in the thermal conductivity with the response functions

As shown in Figure S5, the T_{min} for the minimum thermal conductivity is consistent with the temperature for the thermal expansion and speed of sound minima, and compressibility maxima for all the pressures. Figure S6 shows the fluctuation properties.

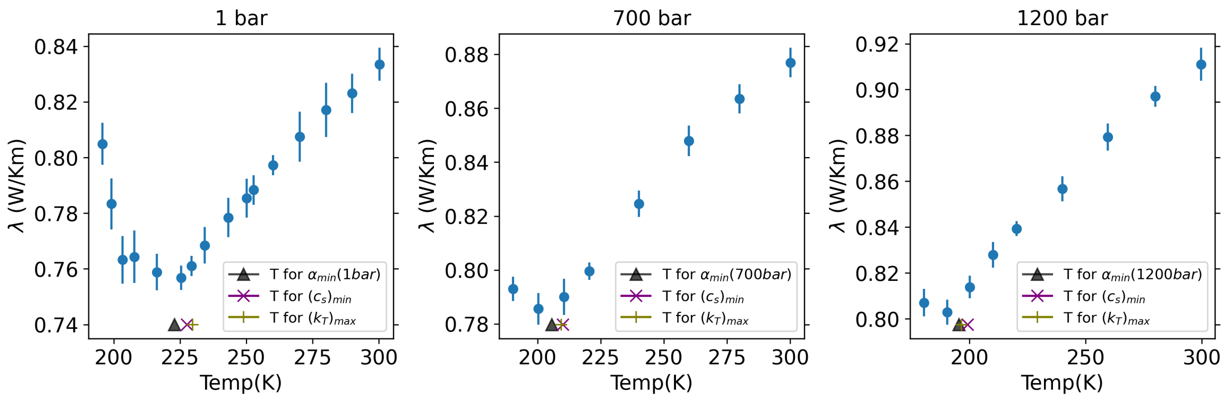

Figure S5: Average thermal conductivity (cricles) for the three isobars (1, 700, 1200 bar). The markers indicate the temperature for the minimum thermal expansion coefficient (△), minimum speed of sound (×), and maximum isothermal compressibility (+).

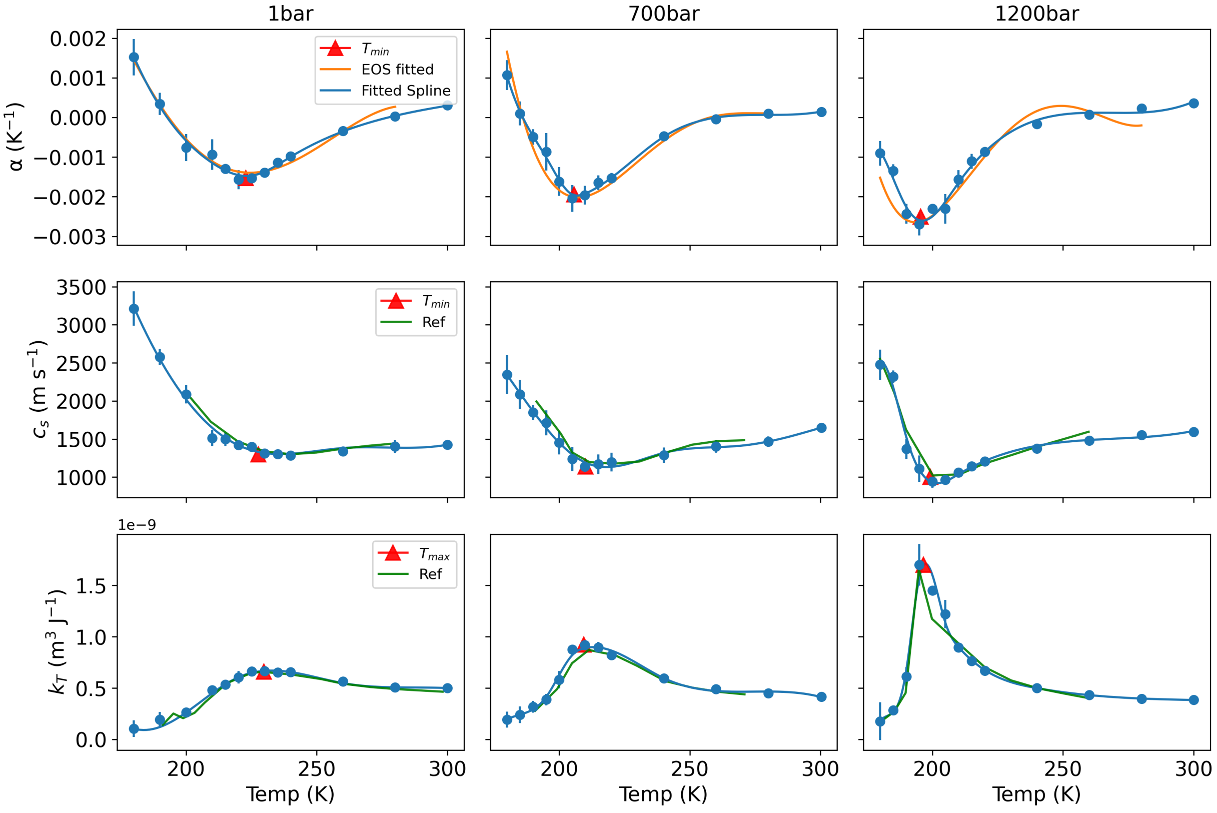

Figure S6: Fluctuation properties: thermal expansion coefficient, isothermal compressibility, and the speed of sound of TIP4P /2005 water calculated from equilibrium simulations as a function of temperature and for the 1, 700, and 1200 bar isobars. The triangle represent the minimum (top two rows) or maximum (bottom row) values for each properrty, obtained from fittings of the data points. The orange curves in the top three panesl show the thermal expansion coefficients calculated from the derivative of the density obtained from NPT simulations, and fitted to a 5th-degree polynomial. The green curves in the other panels represent the speed of sound and compressibility data points taken from previous work.[S11]

5. TIP4P/ICE Simulations

Figure S7 shows the thermal conductivity of TIP4P/ICE at 400 bar and TIP4P/2005 at 1 bar.

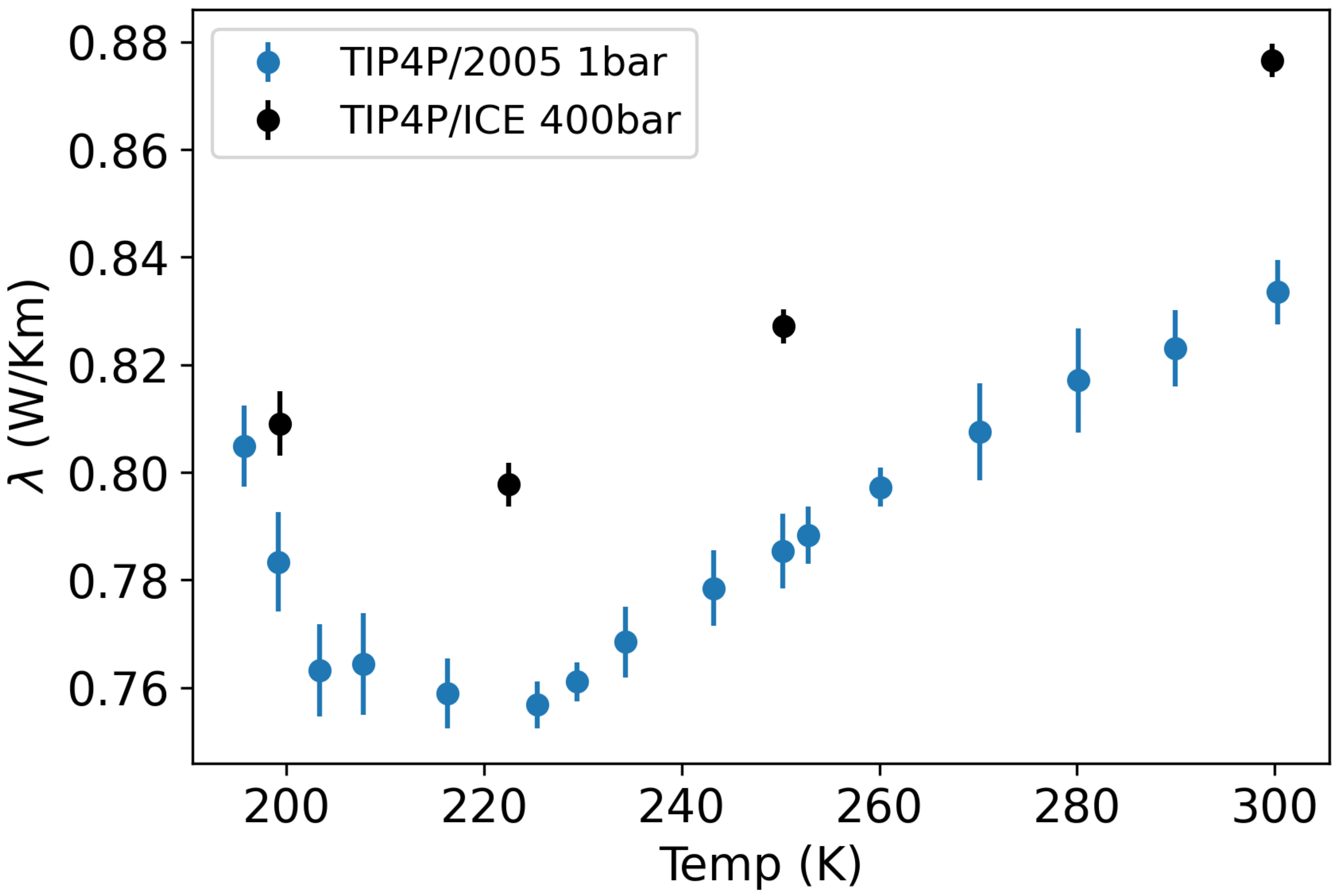

Figure S7: Average thermal conductivity of theTIP4P/ICE model at 400 bar (black circles) compared with the TIP4P/2005 1 bar data (blue circles).

References

(S1) Isele-Holder, R. E.; Mitchell, W.; Hammond, J. R.; Kohlmeyer, A.; Ismail, A. E. Reconsidering Dispersion Potentials: Reduced Cutoffs in Mesh-Based Ewald Solvers Can Be Faster Than Truncation. *Journal of Chemical Theory and Computation* **2013**, *9*, 5412–5420.

(S2) Hockney, R., Eastwood, J., Eds. *Computer Simulation Using Particles*; McGraw-Hill: New York, 1981.

(S3) Bekker, H.; Berendsen, H.; Dijkstra, E.; Achterop, S.; Drunen, R.; van der Spoel, D.; Sijbers, A.; Keegstra, H.; Reitsma, B.; Renardus, M. Gromacs: A parallel computer for molecular dynamics simulations. *Physics Computing* **1993**, *92*, 252–256.

(S4) Allen, M. P.; Tildesley, D. J. *Computer simulation of liquids*; Oxford University Press: London, England, 1987.

(S5) Bussi, G.; Donadio, D.; Parrinello, M. Canonical sampling through velocity rescaling. *The Journal of chemical physics* **2007**, *126*, 014101.

(S6) Bresme, F.; Lervik, A.; Bedeaux, D.; Kjelstrup, S. Water polarization under thermal gradients. *Physical review letters* **2008**, *101*, 020602.

(S7) Chapman, A.; Bresme, F. Polarisation of water under thermal fields: the effect of the molecular dipole and quadrupole moments. *Phys. Chem. Chem. Phys.* **2022**, *24*, 14924–36.

(S8) Abascal, J. L. F.; Vega, C. A general purpose model for the condensed phases of water: TIP4P/2005. *The Journal of chemical physics* **2005**, *123*, 234505.

(S9) Armstrong, J.; Bresme, F. Temperature inversion of the thermal polarization of water. *Phys Rev E Stat Nonlin Soft Matter Phys.* **2015**, *92*, 060103.

(S10) Iriarte-Carretero, I.; Gonzalez, M. A.; Armstrong, J.; Fernandez-Alonso, F.; Bresme, F. The rich phase behavior of the thermopolarization of water: from a reversal in the polarization, to enhancement near criticality conditions. *Phys. Chem. Chem. Phys.* **2016**, *18*, 19894–19901.

(S11) Bresme, F.; Biddle, J. W.; Sengers, J. V.; Anisimov, M. A. Communication: minimum in the thermal conductivity of supercooled water: a computer simulation study. *J Chem Phys.* **2014**, *140*, 161104.
